# Chlorogenic Acids Inhibit Adipogenesis: Implications of Wnt/*β*-Catenin Signaling Pathway

**DOI:** 10.1155/2021/2215274

**Published:** 2021-11-20

**Authors:** Mengting Liu, Jian Qin, Jing Cong, Yubin Yang

**Affiliations:** ^1^The First Affiliated Hospital of Guangdong Pharmaceutical University, Guangzhou 510080, China; ^2^The Seventh Affiliated Hospital of Sun Yat-Sen University, Shenzhen 518000, China; ^3^Academic Department, Giant Praise (HK) Pharmaceutical Group Limited, Changchun 130033, China

## Abstract

In our previous in vitro study, we found that chlorogenic acid (CGA) inhibited adipocyte differentiation and triglyceride (TG) accumulation, but the underlying mechanism is still unclear. Accumulative genetic evidence supports that canonical Wnt signaling is a key modulator on adipogenesis. *Methods*. In this study, 3T3-L1 cells were induced adipogenic differentiation and then treated with CGA. We investigate the effect of CGA in inhibiting adipogenesis and evaluate its role in modulating Wnt10b (wingless integration1 10b), *β*-catenin, glycogen synthase kinase-3*β* (GSK-3*β*), and peroxisome proliferator-activated receptor *γ* (PPAR-*γ*) involved in the Wnt (wingless integration1)/*β*-catenin signaling pathway. *Results*. The result showed that after CGA treatment, lipid accumulation and TG level decreased significantly in 3T3-L1 cells, indicating that CGA could inhibit adipogenesis. In addition, CGA repressed the induction of adipocyte differentiation biomarkers as PPAR-*γ*, adipocyte protein 2 (aP2), fatty acid synthase (FAS), and lipoprotein lipase (LPL), and the secretion of GSK-3*β* in a dose-dependent manner upregulated the expression of *β*-catenin and Wnt10b both in gene and protein levels. Moreover, CGA induced phosphorylation of GSK-3*β* and promoted the accumulation of free cytosolic *β*-catenin in 3T3-L1 adipocytes. *Conclusion*. Overall, these findings gave us the implications that CGA inhibits adipogenesis via the canonical Wnt signaling pathway.

## 1. Introduction

Obesity is becoming a worldwide health concern for its close association with cardiovascular disease, type 2 diabetes, hypertension, metabolic syndrome, osteoarthritis, and certain types of cancer [1]. According to the World Health Organization (WHO), the worldwide prevalence of obesity nearly tripled from 1975 to 2016 [2]. It is also estimated that, by the year 2030, 38% of the world's adult population will be overweight and another 20% will be obese [3]. The development of obesity is characterized by hypertrophy (increase in size) and hyperplasia (increase in number). Hyperplasia, also defined as adipogenesis, is the process by which adipocytes develop from adipose-derived stem cells to lipoblasts, then into preadipocytes, and ultimately into the mature adipocytes through cell differentiation [4]. The inhibition of the early stage of adipogenesis is one of the many possible potential targets for the prevention and/or treatment of obesity [5].

Adipogenesis is triggered by a cascade of transcriptional networks, mainly PPAR-*γ* and CCAAT-enhancer-binding proteins (C/EBPs), along with the regulation of several signaling pathways, including Wnt, mitogen-activated protein kinase (MAPK), Hedgehog, and Insulin-Like Growth Factor 1 (IGF-1) [6]. One of the extracellular signaling pathways recently known to affect adipogenesis is the Wnt/*β*-catenin pathway. The importance of peculiar Wnt/*β*-catenin signaling has directed considerable attention in the future production of therapeutic approaches in obesity [7].

There are many options for treating obesity on the market that are prevented from being widely used in a clinical environment because of their side effects such as hypertension, cardiovascular problems, damage to liver function, and psychiatric disorders [8]. Thus, searching for new antiadipogenic agents from natural materials targeting these pivotal adipogenic genes might be a promising method for the prevention of obesity [4].

**Figure 1:**
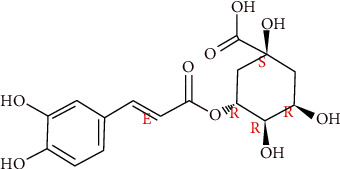
Structure of CGA (http://www.chemexper.com/).

CGA (Figure 1), a phenolic compound from the hydroxycinnamic acid family [9], widely exists in medicinal herbs. It has been shown to have a strong antioxidant, anti-inflammatory, antilipidemic, and antihypertensive effect [10–12]. Furthermore, an accumulation of studies has evaluated the health benefits of CGA on obesity, diabetes, and hypertension in both clinical studies and animal researches [13–15].

The 3T3-L1 cell line has been used extensively and reliably in in vitro models for studying the conversion of preadipocytes into adipocytes [16]. Several studies have reported that CGA exerts antiobesity effects by inhibiting the differentiation and lipogenesis of 3T3-L1 cells [17]. Our previous data indicated that CGA inhibits triglyceride accumulation during adipogenesis of 3T3-L1cells. However, the mechanism responsible for these effects remains unclear. Consequently, the aim of this study is to investigate the underlying mechanism of CGA on adipocyte differentiation and adipogenesis and explore the potential molecular mechanisms.

## 2. Materials and Methods

### 2.1. Chemicals and Reagents

Dulbecco's modified eagle medium (DMEM, Hyclone, South Logan, UT, USA, Cat. No. SH30022.01B), penicillin-streptomycin (Hyclone, South Logan, UT, USA, Cat. No. SH30010), Cell Counting Kit-8 (CCK-8) solution (Dojindo, Kumamoto, Japan, Cat. No. C0037), dimethyl sulfoxide (DMSO, Sigma, St. Louis, MO, USA, Cat. No. I-6481), Phosphate-Buffered Saline (PBS, Hyclone, South Logan, UT, USA, Cat. No. SH30256.01B), 3-isobutyl-1-methylxanthine (IBMX, Sigma, St. Louis, MO, USA, Cat. No. I-7018), Dexamethasone (Sigma, St. Louis, MO, USA, Cat. No. D-4902), Insulin (Sigma, St. Louis, MO, USA, Cat. No. I-5500), fetal bovine serum (FBS, Gibco, Rockville, MD, USA, Cat. No. 10270-106), CGA (Chengdu Must Bio-Technology CO., LTD, Chengdu, China), lithium chloride (LiCl, Chengdu Must Bio-Technology CO., LTD, Chengdu, China), and Oil Red O (ORO, Beijing Leagene Biotechnology, CO., LTD, Beijing, China) were used. Polyclonal antibodies against PPAR-*γ*, aP2, FAS, LPL, and GSK3*β* were obtained from cell signaling (Danvers, MA, USA).

### 2.2. Cell Culture and Differentiation

The 3T3-L1 cells were obtained from the Sojubio (Guangzhou, China). This cell line has been used to study physiopathological mechanisms of adipogenesis [16]. Primary cells were passaged more than 3 times, and the cells were used for subsequent experiments. To induce adipogenesis, the 3T3-L1 cells were seeded at a density of 1 × 10^4^ cell/cm^2^ in 24-well plates. Following a 48 h culture after cells reached 90–95% confluence (Day 0), the cells were treated with adipogenic medium containing DMEM with 10% FBS, 0.25 mM 3-isobutyl-1-methylxanthine (IBMX), 1 *µ*M dexamethasone, and 500 nM insulin for 2 days (Day 2). And then, cells were maintained in 10% FBS/DMEM with 500 nM insulin for an additional 4 days (Day 6). Thereafter, the cells were maintained in postdifferentiation medium containing 10 g/mL insulin in 10% FBS/DMEM. Differentiation was measured by the appearance of lipid droplets with the use of the dye ORO. Cells were maintained at 37°C in a humidified 5% CO_2_ and differentiated for a total of 12 days with medium changed every 2 days.

### 2.3. Cell Viability

The nontoxic concentrations ranging from 1 to 500 *µ*M previously reported by researchers were evaluated for CGA in the present study [14]. The influence of the CGA on cellular viability was determined by the CCK-8 assay. 3T3-L1 preadipocytes were seeded in 24-well plates and cultured to 100% confluence in culture medium (Day 0). Then, different concentrations at 0, 50, 100, 200, and 240 *µ*g/mL of CGA were added to the media on Day 0. The cell viability of the extracts was determined at 12, 24, and 48 h. CCK-8 solution at 10% was added to each well, and the plates were incubated for 4 h at 37°C. After incubation, absorbance was read at 450 nm as optical density (OD) value by Multiskan Microplate Photometer (FC) enzyme-labeled instrument Multiscan MK3 (Thermo Fisher, Waltham, MA, USA), and the surviving cell fraction was calculated. The cell viability by OD values and the percentage of viable cells following treatment with the CGA compared to the vehicle control (0.1% DMSO) is expressed as follows.

1. The proliferation rate = average OD value of certain time point/average OD value of Day 0 × 100%
2. The inhibition rate = (1 − average OD value of detected samples/average OD value of control group) × 100%

### 2.4. Oil Red O Staining

3T3-L1 cells were washed with PBS three times and fixed for 10 min in 4% paraformaldehyde. Fixed cells were stained with the ORO-isopropanol at 3 mg/mL in 60% (v/v) isopropyl alcohol for 1 h at 25°C, and the excess stain was washed by 70% ethanol and water. Cells were visualized by using an IX-73 inverted biomicroscope (Olympus Corporation, Tokyo, Japan) to evaluate stained lipid droplets. The diameters and areas of lipid droplets were analyzed by the Image pro plus 6.0 (Media Cybernetics, Rockville, MD, USA).

### 2.5. Intracellular Lipid Content Assay

Adipogenesis was assessed in the well-characterized mature 3T3-L1 cell models by measuring the accumulation of TG as described previously using Triglyceride Assay Kit (Nanjing Jiancheng Bio-engineering Institute, Nanjing, China) according to the manufacturer's instruction.

### 2.6. Immunofluorescence Analysis

Cells were fixed in 4% paraformaldehyde for 30 min and permeabilized in 0.2% Triton X-100 for 5 min. After blocking with goat serum for 30 min, the cells were incubated overnight at 4°C with diluted monoclonal mouse anti-*β*-catenin antibody (PTG, Rosemont, PA, USA) overnight. After washing three times with PBS, cells were incubated with Rhodamine (TRITC)-conjugated goat anti-rabbit IgG (*H* + *L*) (Thermo und Life, New York, NY, USA) at 1:200 for 1 h at room temperature in the dark and then stained with 4′, 6-diamidino-2-phenylindole (DAPI; Sigma-Aldrich, St. Louis, MO, USA) for 10 min. Slides were then washed three times and mounted. Samples were viewed with a Leica confocal microscope (Leica DMI6000B, Nussloch, Germany).

### 2.7. Real-Time Polymerase Chain Reaction (RT-PCR)

The total Ribonucleic Acid (RNA) was extracted from cells using TRIzol reagent (Invitrogen^TM^, Carlsbad, CA, USA), according to the manufacturer's instructions. The sequence and GenBank accession number for the forward and reverse primers used to quantify mRNA are listed in Table 1. The reaction mixture had a final volume of 10 *µ*L containing 0.5 mM of all the primers. Briefly, first-strand complementary deoxyribonucleic acid (cDNA) was synthesized from 0.2 *µ*g of total RNA using M-MLV Reverse Transcriptase (RevertAid^TM^, Waltham, MA, USA). The total reaction volumes of RT-PCR reactions were 20 *µ*L. The system consisted of 2 *µ*L RNA, 1 *µ*L Oligonucleotide 18 primer, 9 *µ*L RNase-free water, 4 *µ*L 5× reaction buffer, 1 *µ*L Riblock Rnase Inhibitor, 2 *µ*L 10 mM deoxyribonucleoside triphosphate mixture (dNTP MIX), and 1 *µ*L Moloney Murine Leukemia Virus reverse transcriptase (MMulV). The reaction products were stored at −80°C for long-term preservation. The cDNA was amplified in QuantiFast SYBR Green RT-PCR Kit (Roche^TM^, Basel, Switzerland) in a Bio-Rad iCycler iQ Real-Time PCR System (Invitrogen^TM^, Carlsbad, CA, USA). The sequence and GenBank accession number for the forward and reverse primers used to quantify mRNA are listed in Table 1. The reaction mixture had a final volume of 10 *µ*L containing 0.5 mM of all the primers. The mixture was subjected to the following program of amplification: the first segment of 10 min at 95°C for preincubation of the enzyme, the second segment of amplification consisting of 35 or 40 cycles with each cycle consisting of a thermal ramp to 20°C/s, an alignment segment to 61°C with 7 s thermal ramp to 20°C/s, and an amplification segment at 72°C for 10 s with a thermal ramp of 20°C/s. The program consisted of an initial denaturation at 95°C with a thermal ramp of 20°C/s, realignment at 65°C for 15 s with a thermal ramp of 20°C/s, and finally, a slow denaturation to 95°C at a thermal ramp of 0.1°C/s, with continuous measurement of the fluorescent signal integrity of PCR products that were verified by electrophoresis in 2% agarose gel stained with ethidium bromide (Sigma^TM^, St. Louis, MO, USA).

The statistical analysis of the qRT-PCR was obtained by using the comparative threshold-cycle (CT) method, which calculates the relative changes in gene expression levels of the target gene normalized to the housekeeping gene glyceraldehyde-3-phosphate dehydrogenase (GAPDH). The relative quantification (2^∆∆Ct^) method was used to analyze the data. Each experiment was repeated independently three times. Results are reported as a fold change of the value obtained for the vehicle control.

### 2.8. Western Blotting

After 12 days of differentiation, cells were removed from the plates, and the protein expression of the key molecules was evaluated by Western blotting. Cells were washed in ice-cold PBS and then were resuspended in lysis buffer containing 50 mM Tris-HCl (pH 8.0), 0.4% Nonidet P-40, 120 mM NaCl, 1.5 mM MgCl_2_, 0.1% sodium dodecyl sulfate (SDS), 2 mM phenylmethylsulfonyl fluoride, 80 *µ*g/mL leupeptin, 3 mM NaF and 1 mM dithiothreitol (DTT), and a protease inhibitor cocktail to obtain the total cell lysates. The total cell lysates were centrifuged at 12,000 rpm for 20 min at 4°C to remove insoluble materials. Protein extract samples (20 *μ*g) were separated by 10% SDS-polyacrylamide gel electrophoresis (SDS-PAGE) and transferred onto polyvinylidene difluoride (PVDF) membranes at 150 mA for 1 h. The membranes were then blocked for 1 h at room temperature with phosphate-buffered saline (PBS) containing 5% skim milk and 0.1% Tween 20. The membranes were then incubated with 1:1000 dilution of primary antibodies overnight at 4°C and subsequently with a horseradish peroxidase-conjugated anti-rabbit secondary antibody for 1 h at room temperature. The protein bands were detected with an enhanced chemiluminescence (ECL) detection system (Genecopoeia, Rockville, MD, USA) according to the manufacturer's instructions and visualized with the G:BOX EF imaging system and the Gene Snap program (Genecopoeia, Rockville, MD, USA). The Western blotting results were confirmed twice by independent experiments.

### 2.9. Statistical Analysis

All experiments were repeated independently at least 3 times. All quantitative data are presented as means ± SEM. Statistical significance for comparisons between treated samples and corresponding untreated samples was performed using Statistical Product and Service Solutions (SPSS) 22.0 (SPSS, Chicago, IL, USA). Student's *t*-test and one-way analysis of variance (ANOVA) followed by the post hoc Bonferroni test were used. A *P* value of less than <0.05 was considered statistically significant.

## 3. Results

### 3.1. Effect of CGA on the Cell Viability

**Figure 2:**
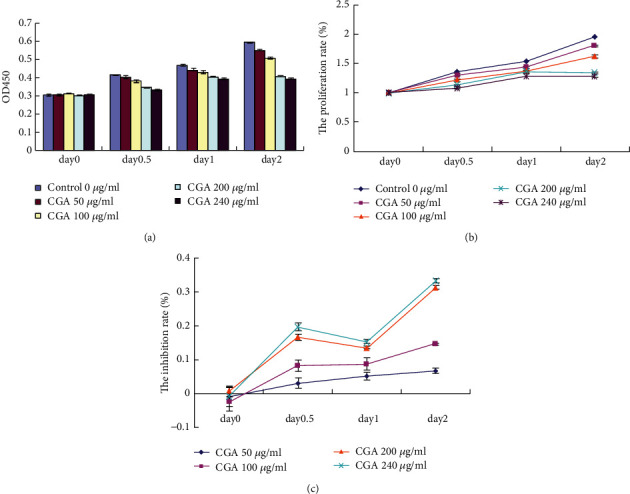
Cell toxicity test on different concentrations of CGA on 3T3-L1 cells.

The impact of CGA on cell viability was determined by the absorbance of cell suspension as optical density value (OD) value by Cell Counting Kit-8 (CCK-8) assay. 3T3-L1 cells incubated for 12, 24, and 48 h with CGA do not cause significant loss of cell viability at concentrations ranging from 50 to 240 *μ*g/mL. 240 *μ*g/mL CGA slightly decreased the proliferation rate and increased the inhibition rate of 3T3-L1 cells from Day 1 to Day 2 (Figure 2).

### 3.2. CGA Repressed Lipid Accumulation of 3T3-L1 Cells

**Figure 3:**
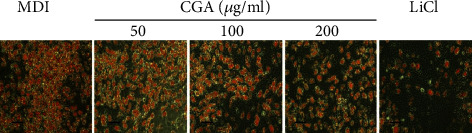
Representative ORO staining of different groups in 3T3-L1cells induced for 7 days. Scale bar: 200 *μ*m.

We further investigated the effect of CGA on lipid accumulation by adding CGA (0, 50, 100, and 200 *μ*g/mL) and lithium chloride (LiCl) of 20 mM separately to the dimethyl sulfoxide (DMSO) and MDI (methylisobutylxanthine, dexamethasone, and insulin) induction medium of cells on Day 0 of differentiation. The final concentration of DMSO was kept at 0.1% throughout the differentiation period. Oil Red O (ORO) staining was applied in adipocytes-induced differentiation for 7 days (Figure 3). We analyzed the diameters and areas of lipid droplets in ORO staining by using the Image pro plus 6.0 (Table 2). Compared to the negative control MDI group, 100 and 200 *μ*g/mL CGA significantly reduced the areas and diameters of lipid droplets (^*∗*^*P* < 0.05); however, compared with the MDI group, 50 *μ*g/mL CGA did not change the areas and diameters of lipid droplets (^*∗∗*^*P* > 0.05). These data indicate that 50 *μ*g/mL CGA had almost no impact on them. Based on these, our in vitro study was focused on the concentration range of 100 and 200 *μ*g/mL CGA.

### 3.3. CGA Decreased the Intracellular TG Content of 3T3-L1 Cells

**Figure 4:**
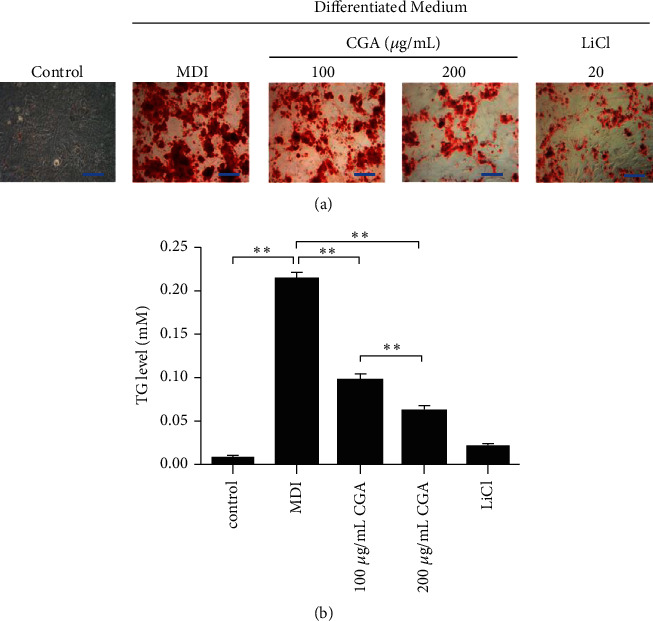
Effects of CGA on the adipogenesis of 3T3-L1 cells induced for 12 days. (a) Intracellular lipid droplets were stained with Oil Red O.Scale bar: 200 *μ*m. (b) Intracellular triglyceride concentrations. Data are presented as the mean ± SE from three independent experiments. ^*∗*^*P* < 0.05, ^*∗∗*^*P* < 0.01.

On Day 0 of differentiation, we separately added CGA (100, 200 *μ*g/mL) and 20 mM LiCl to the DMSO with MDI induction media of cells. As the undifferentiated group, the vehicle control was cultured in the 0.1% DMSO. By inducing differentiation for 12 days, 95% 3T3-L1 cells were characterized with increasing numbers and sizes of lipid droplets as the mature adipocyte phenotype. On Day 12, the intracellular lipid accumulation and lipid droplets are greater in the MDI group as compared to those of vehicle control (0.1% DMSO). 200 *μ*g/mL CGA resulted in a greater reduction in intracellular lipid content formation compared to that treated with 100 *μ*g/mL CGA (Figure 4(a)).

On Day 12, the total cellular TG content of adipocytes was evaluated. Consistent with ORO staining, Triglyceride Assay showed a very low level of TG was present in the medium of vehicle control and increased approximately 20 times in the medium of MDI group (Figure 4(b), *P* < 0.01). CGA downregulated the TG contents in a dose-dependent manner, 200 *μ*g/mL CGA had a more notable effect on the reduction of TG content as compared with 100 *μ*g/mL CGA (Figure 4(b), *P* < 0.01).

### 3.4. CGA Suppressed the Expression of Genes and Proteins Associated with Adipogenesis and Lipid Accumulation During Differentiation of 3T3-L1 Cells

**Figure 5:**
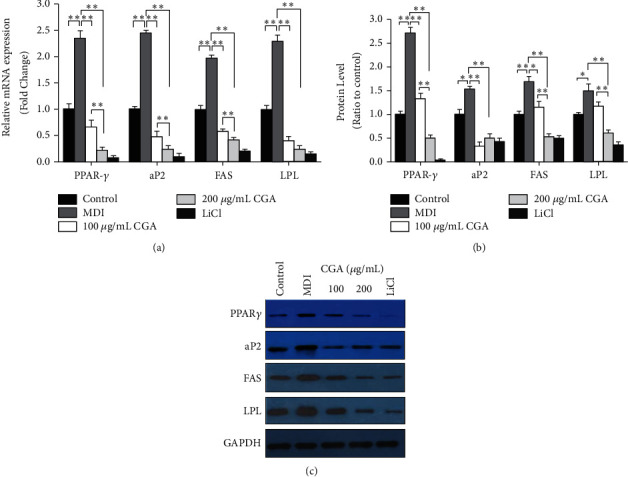
Effects of chlorogenic acid on the expression of transcription factors and genes associated with adipogenesis in 3T3-L1 cells. (a, b) Protein expression of PPAR-*γ*, aP2, FAS, and LPL by Western blot analyses. Data are presented as means ± SE from three independent experiments. ^*∗*^*P* < 0.05, ^*∗∗*^*P* < 0.01. (c) Effects of CGA on gene expression of PPAR-*γ*, aP2, FAS, and LPL in 3T3-L1 cells. Data are presented as means ± SE from three independent experiments. ^*∗*^*P* < 0.05, ^*∗∗*^*P* < 0.01.

Compared to vehicle control, the gene and protein expression of PPAR-*γ*, aP2, LPL, and FAS was markedly upregulated in the MDI group, while they were decreased by CGA (100 and 200 *μ*g/mL) in a dose-dependent manner by comparison with the MDI group (Figures 5(a)–5(c)). These results indicate that CGA affects lipid accumulation by regulating the expression of related genes.

### 3.5. CGA Stimulated the Expression of the Wnt/*β*-Catenin Cascade

**Figure 6:**
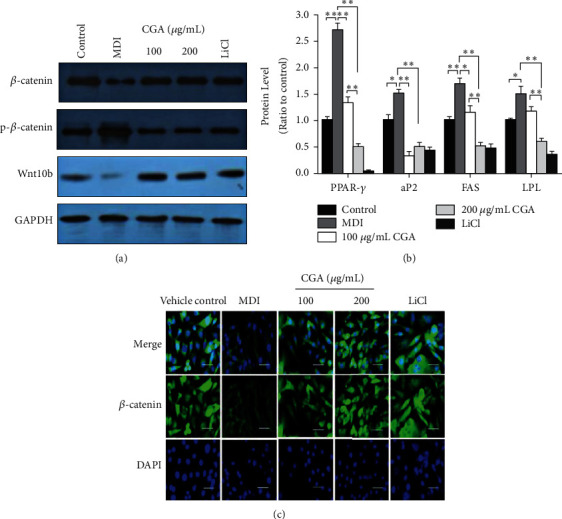
CGA activates the expression of the Wnt/*β*-catenin signaling pathway components. (a) Protein expression of *β*-catenin, non-p-*β*-catenin, and Wnt10b by Western blot analysis in 3T3-L1 cells. (b) Gene expression of *β*-catenin, non-p-*β*-catenin, and Wnt10b in 3T3-L1 cells. Data are presented as means ± SE from three independent experiments. ^*∗*^*P* < 0.05, ^*∗∗*^*P* < 0.01. (c) The expression of *β*-catenin in 3T3-L1 cells was increased in the groups treated with CGA and LiCl compared with the MDI group, detected by immunofluorescence assay. *β*-catenin staining is shown as green fluorescence. DAPI staining is shown as blue fluorescence. Scale bars, 100 *μ*m.

Wnt/*β*-catenin cascade has been shown to inhibit adipogenesis by regulating various cellular events including mesenchymal cell fate determination and differentiation [7]. To further elucidate the molecular mechanism underlying CGA-mediated suppression of adipogenesis, we examined key molecules of the Wnt/*β*-catenin cascade. CGA increased *β*-catenin and Wnt10b expression as compared with those of the MDI group. In addition, *β*-catenin and Wnt10b were expressed at much higher levels in the 200 *μ*g/mL CGA group than those in the 100 *μ*g/mL CGA group (Figures 6(a)-6(b)).

When the canonical Wnt/*β*-catenin signaling pathway is activated by the Wnt ligands, *β*-catenin is prevented from phosphorylation and translocates into the nucleus where it stimulates the transcription of downstream genes [18]. In this research, the effect of CGA on the free cytosolic *β*-catenin was assessed by immunofluorescence assay. Immunofluorescence labeling *β*-catenin revealed that *β*-catenin was decreased dramatically in the MDI group compared with the vehicle control. Noteworthily, treatment by CGA and LiCl restored the free cytosolic *β*-catenin. These findings showed that the level of free cytosolic *β*-catenin increased after CGA treatment during adipogenic differentiation in 3T3-L1 cells (Figure 6(c)).

### 3.6. CGA Suppressed Glycogen Synthase Kinase-3*β* (GSK3*β*) in the Wnt/*β*-Catenin Signaling Pathway

**Figure 7:**
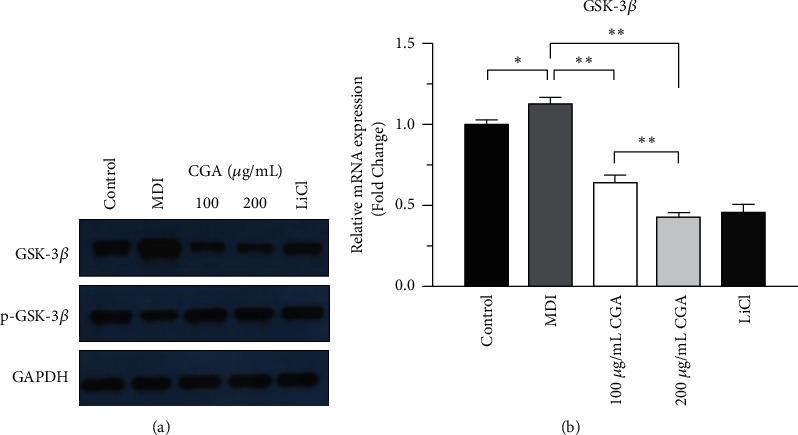
Activation of the Wnt/*β*-catenin signaling pathway by chlorogenic acid targeting GSK3*β*. (a) The protein expression of phospho-GSK3*β* and total GSK3*β* by Western blotting. (b) The gene expression of GSK3*β* in 3T3-L1 cells. Data are presented as means ± SE from three independent experiments. ^*∗*^*P* < 0.05, ^*∗∗*^*P* < 0.01.

Previous studies demonstrated that GSK3*β* phosphorylates *β*-catenin, leading to its proteasomal degradation in the absence of Wnt activation. Conversely, suppressing of GSK-3*β* is associated with stabilization of *β*-catenin which allows the accumulation of *β*-catenin in the cytoplasm and entry into the nucleus [19]. In this research, upon inducing 3T3-L1 cell differentiation, Western blotting results demonstrated that the protein expression of total GSK-3*β* was increased and p-GSK-3*β* was decreased in the MDI group compared with the vehicle control group. In contrast, CGA inhibited endogenous GSK-3*β* protein expression but induced phosphorylation of GSK-3*β* in 3T3-L1 preadipocytes compared with the MDI group (Figure 7(a)). This result was consistent with RT-PCR outcomes (Figure 7(b)).

## 4. Discussion

Though the U.S. Food and Drug Administration (FDA) has approved several drugs such as Qsymia, Belviq, Contrave, and Saxenda for the treatment of obesity, the use of these drugs has been associated with side effects, including the development of cardiovascular disease and adverse reactions of the gastrointestinal tract [20]. Researchers have been exerting great effort to explore safe and effective medicaments with less side effects. Therefore, natural products that have antiobesity effects have been widely investigated in recent years [21].

Suppression of lipogenesis and adipogenesis is a feasible approach against obesity, and lipid accumulation reflected the process of differentiation of preadipocytes into adipocytes [22]. Consistent with similar research [23], in the present study, we observed that CGA inhibited adipogenesis and decreased fat deposition as measured by ORO staining. Meanwhile, according to the TG assay, CGA inhibits the fat accumulation in 3T3- L1 cells in a dose-dependent manner (Figures 3 and 4), indicating the potent antiobesity activity of CGA. Lithium was originally approved as a safe medicine for the treatment of mental disorders. Interestingly, data from previous researches showed that LiCl (a GSK3*β* inhibitor) can inhibit adipocyte differentiation [24]. In this study, we set LiCl treatment as a positive control group. As the agonist of Wnt/*β*-catenin signaling, LiCl can significantly inhibit adipogenesis by acting on the Wnt signaling pathway [25]. 20 mM treatment concentration is considered to be the optimal concentration.

PPAR-*γ* is a ligand-activated factor and the most important transcriptional modulator of adipocyte development in all types of adipose tissue by mediating the expression of target genes as aP2, FAS, and LPL [26]. In the present study, we observed that CGA dose-dependently downregulated both the mRNA and protein levels of PPAR-*γ* in MDI-treated 3T3-L1 cells. Meanwhile, the expression of target genes aP2, FAS, and LPL was also decreased in a dose-dependent manner after CGA treatment (Figure 5). Similar to our results, a recent study has also shown that CGA inhibited 3T3-L1 cell differentiation in the middle and late stages and reduced the fat accumulation by affecting the transcriptional activity of PPAR-*γ* [23]. However, another research has documented that CGA increased the expression of mRNA PPAR-*γ* in 3T3-L1 adipocytes [14]. The differences between the latter research with ours lie in the fact that, firstly, the latter research just tested the mRNA of PPAR-*γ* but no protein level. Secondly, it only investigated the single dosage of CGA at 50 *μ*M, while this dosage was regarded as ineffective in suppressing lipid accumulation in our present study. However, it does not mean that the outcome of these two researches conflicts with each other, but further investigation of CGA at 50 *μ*M on protein and mRNA of PPRA-*γ* in vivo and in vitro should be done to clarify this problem. Taken together, our results demonstrate that CGA inhibited adipocyte differentiation and adipogenesis through downregulation of the transcription factor gene PPAR-*γ* and related target genes (aP2, FAS, and LPL) in the 3T3-L1 cells.

Wnt family proteins can inhibit preadipocyte differentiation through a canonical pathway (*β*-catenin dependent) and noncanonical (*β*-catenin independent). Canonical Wnt signaling has been explored more thoroughly than noncanonical signaling [27]. Considerable evidence showed that Wnt/*β*-catenin signaling plays differential regulatory roles in adipogenesis by suppressing the differentiation of preadipocyte cells to mature adipocytes. It was demonstrated that the Wnt/*β*-catenin signaling is similar to an adipogenic switch—when it is on, adipogenesis is repressed; when it is off, adipogenesis is initiated [28]. As the central protein of canonical Wnt signaling, *β*-catenin is particularly crucial in that the cytoplasmic accumulation and then nuclear translocation of *β*-catenin (nonphosphorylated *β*-catenin) enhance the transcriptional activity and regulate the target genes expression [29]. In the current study, we investigated whether the antiadipogenic activity of CGA in adipocyte differentiation involves canonical Wnt signaling. Some natural products have been reported to exert pharmacological antiobesity effects by targeting the Wnt/*β*-catenin pathway [30, 31]. Our results align with these findings. Immunofluorescence staining showed that *β*-catenin was mainly located in the cytoplasm rather than the nucleus in differentiated adipocytes, indicating that the Wnt/*β*-catenin pathway was inactive, while CGA promoted the nuclear translocation of *β*-catenin from the cytoplasm (Figure 6(c)). Consistently, Western blotting showed that the protein levels of total *β*-catenin and non-p *β*-catenin were upregulated after CGA treatment (Figures 6(a), and 6(b)), suggesting the increased *β*-catenin nuclear translocation by stimulation of CGA. As a key negative regulator of *β*-catenin in the Wnt/*β*-catenin signaling pathway, GSK3*β* induces aminoterminal serine-threonine phosphorylation of *β*-catenin, which is tightly regulated by the complex of *β*-catenin/Axin/GSK-3*β*/APC (adenomatous polyposis coli)/CK1*α* (Casein Kinase1*α*), so that *β*-catenin in the cytoplasm remains at a relatively low level [32]. Therefore, the phosphorylation of GSK-3*β* leads to the disintegration of the complex and the nondegradation of *β*-catenin. After the accumulation in the cytoplasm, *β*-catenin enters the nucleus and upregulates the transcription of genes [19]. In the present study, we found that CGA increased the phosphorylation level of GSK-3*β*, suggesting that CGA might inhibit adipocytes and lipid accumulation by activating the *β*-catenin signaling pathway via inducing GSK-3*β* phosphorylation. This is the first study showing the mechanism of CGA inhibiting adipocyte differentiation and its effects on the Wnt/*β*-catenin signaling pathway after adipocyte differentiation.

## 5. Conclusions

Our findings suggest that CGA may be a potential therapeutic agent for obesity with the mechanism involving the Wnt/*β*-catenin signaling pathway. As the preliminary study, this research investigated the underlying mechanism of the inhibitory effect of CGA on adipogenesis via the Wnt/*β*-catenin signaling pathway in vitro. Further studies will be needed in vivo using gene knockout animal models to clarify the mechanism based on the Wnt/*β*-catenin signaling pathway.

## Figures and Tables

**Table 1:** Primers used for real-time PCR.

Gene	Forward primer (5′ to 3′)	Reverse primer (5′ to 3′)
aP2	GAGCATCATAACCCTAGATGG	GCTCATGCCCTTTCATAA AC
FAS	CTATACTACCCAAGACAGGAAC	CAGCTCCTTGTATACTTCTCC
LPL	GTAGTAGACTGGTTGTATCGG	GTAGTTAAACTCCTCCTCCATC
PPAR-*γ*	CCTTCAGTTCACTCTCAGTAAG	CACACTCTATGTCACTCCATAC
*β*-catenin	GTGGATTCTGTACTGTTCTACG	CTTCTCATAAGTGTAGGTCCTC
Wnt10b	TTGTGGATCCTGCACCTGAA	GTTAGAGCCCGACTGAACAAA
GSK-3*β*	ACCCTCATTACCTGACCTT	TCGGCAGACAATTCAACTC
GAPDH	GGCCTCCAAGGAGTAAGAAA	GCCCCTCCTGTTATTATGG

**Table 2:** Parameters of lipid droplets (mean ± SEM).

Group	Number of lipid droplets	Total area (*μ*m^2^)	Mean area (*μ*m^2^)	Mean diameter (*μ*m)
MDI 50 *μ*g/ml	584	184980	162.18 ± 26.21	13.66 ± 4.21
CGA 100 *μ*g/ml	516	189911	158.98 ± 26.75^*a*^	13.58 ± 4.39^*a*^
CGA 200 *μ*g/ml	558	153113	121.71 ± 12.79^*b*^	10.77 ± 5.06^*b*^
CGA	338	34268	101.38 ± 17.17^*b*^	9.34 ± 4.74^*b*^
LiCl	110	8079	73.45 ± 23.67^*b*^	8.28 ± 3.00^*b*^

^*a*^ *P* > 0.05 vs. no significant difference compared with the MDI group. MDI group, ^*b*^*P* < 0.05 significantly different compared with the MDI group.

## Data Availability

The datasets used or analyzed during the current study are available from the corresponding author on reasonable request.
